# Medical Image Retrieval Using Empirical Mode Decomposition with Deep Convolutional Neural Network

**DOI:** 10.1155/2020/6687733

**Published:** 2020-12-26

**Authors:** Shaomin Zhang, Lijia Zhi, Tao Zhou

**Affiliations:** School of Computer Science and Engineering, North Minzu University, Yinchuan 750021, China

## Abstract

Content-based medical image retrieval (CBMIR) systems attempt to search medical image database to narrow the semantic gap in medical image analysis. The efficacy of high-level medical information representation using features is a major challenge in CBMIR systems. Features play a vital role in the accuracy and speed of the search process. In this paper, we propose a deep convolutional neural network- (CNN-) based framework to learn concise feature vector for medical image retrieval. The medical images are decomposed into five components using empirical mode decomposition (EMD). The deep CNN is trained in a supervised way with multicomponent input, and the learned features are used to retrieve medical images. The IRMA dataset, containing 11,000 X-ray images, 116 classes, is used to validate the proposed method. We achieve a total IRMA error of 43.21 and a mean average precision of 0.86 for retrieval task and IRMA error of 68.48 and F1 measure of 0.66 on classification task, which is the best result compared with existing literature for this dataset.

## 1. Introduction

Imaging through different kinds of medical devices plays a fundamental role in clinical diagnosis [1], treatment planning [2], and treatment response assessing [3] in the process of medical care. In modern hospitals, different modalities and protocols of digital imaging techniques have been used to generate diagnostic images for each patient, including computed tomography (CT), X-ray, ultrasound, hybrid positron emission tomography and computed tomography (PET-CT), and magnetic resonance imaging (MRI). These medical images with multiple dimensions (e.g., 2D, volumetric 3D, and time series) reflect anatomic and functional aspects of organs and tissue types that require domain experts' analysis and interpretation. These volumes are usually formed in the Digital Imaging and Communications in Medicine (DICOM) format and stored in picture archiving and communication systems (PACS) [4]. A domain expert can search PACS through patient's ID, study ID, time range, or other textual keywords, which is labor intensive and time consuming. As an important part of computer-aided diagnostics (CAD), content-based medical image retrieval (CBMIR) [5–8] can retrieve medical images mainly via visual contents (e.g., same modality, same body orientation, same anatomical region, or same disease condition) in an existing dataset for more accurate comparative diagnosis.

In the CBMIR domain, there are two major directions in research works. One kind of methods focuses on automatic retrieving images from PACS-like databases, which search images of the same imaging modality, body orientation, body region, and the like [9–11]. Another kind of methods put their efforts into retrieving images that characterize the similar disease convenient for diagnostic comparing [12, 13]. In this study, we follow the former methods to propose an effective CBMIR system for 2D slice retrieval. That is because volumetric 3D medical images are formed by a series of 2D slices acquired from the target body organ, and physicians mainly rely on these 2D slices when they are analyzing and interpreting images on hand [8].

Unlike similarity defined in generic image retrieval domain, the retrieved medical images by directly comparing features using some similarity measure may not be in accordance with what a physician would want for diagnosis, which formed a “semantic gap” in medical image retrieval [5]. To reduce this gap, CBMIR systems are generally designed under a classification-driven strategy. That is, a CBMIR system is trained using supervised approaches with labeled images. When a query image is submitted to the CBMIR system, the query image is classified first, and then, some visual features and similarity measures are used for similarity retrieval [9, 10, 14]. Deep learning is a breakthrough in machine learning research. Using artificial neural networks with many hidden layers to represent digital images has been proven to be a very effective method to describe low-level, mid-level, and high-level semantic features of an image for recognition and other purposes [15–17]. Among different deep learning architectures, deep convolutional neural networks (CNNs) have proven to be powerful tools that achieved very high precision results in many natural image classification contests [18–20]. In the medical field, deep CNNs are also quickly applied for different tasks, and promising results are emerging [9, 10, 21–24]. Training deep CNNs need a large number of labeled images to choose the huge number of parameters. Note that in the medical domain, such large image datasets are quite rare, due to the unbearable high cost of domain experts' manual image labeling and annotations [5, 21, 24]. And in contrast to generic image databases, medical image datasets usually are unbalanced because of uneven incidence rates of different malignancies. Dropout [25], data augmentation, and transfer learning [26] are the most common techniques used to prevent overfitting in the process of training deep CNNs on small and unbalanced image datasets. However, for medical image analysis tasks, these techniques meet various problems [5, 9, 24]; the requirement for a more effective and more robust CBMIR system is still urgent.

In this paper, inspired by pioneering research works [9, 10, 14], we focus on 2D medical image retrieval and put forth an effort to alleviate the two main difficulties in CBMIR (i.e., (1) the labeled medical image datasets are commonly not large enough for training deep CNNs and (2) the imbalance problem is naturally attached to medical image datasets from clinic diagnosis). A new deep CNN-based 2D medical slice retrieval method is proposed, which can be effectively trained on relatively small labeled and unbalanced medical image dataset and promote the retrieval precision. First, in addition to commonly used methods for training deep CNNs on small and unbalanced datasets, e.g., dropout [25] and data augmentation, we supplement nonlinear components by using empirical mode decomposition (EMD) on 2D medical images to enhance effective information and reduce the image noise for training deep CNNs. Second, as for deep CNN architecture in this work, we employ residual network (ResNet) [19] as the backbone network adapted for learning different level features from medical images, which is combined with an attention mechanism to focus on the most relevant features by integrating local and global features in different scales [27]. And center loss function is combined with softmax loss function as a supervision signal in a deep CNN training process to facilitate nearest-neighbor similarity retrieval performance. The contributions of this paper are given as follows:

1. Nonlinear empirical mode decomposition on 2D medical images is proposed for supplementing effective information to original 2D medical images for better distinctively expressing 2D medical images
2. A residual network-based deep CNN model with attention and center loss modules is employed and trained on publicly available medical image datasets. The learned concise feature vectors are suitable for both classification-based and nearest-neighbor similarity-based medical image retrieval and show the great potential to handle large-scale medical image retrieval

## 2. Related Work

Among CBMIR literatures, there are two crucial factors that determine the performance of systems: (1) *Feature vector construction*: medical image features such as texture, shape, etc., should be extracted and formed into a vector to represent the query image and the images in datasets. (2) *Retrieval strategy*: classification-based retrieval strategy, nearest-neighbor search strategy, or their combination should be carefully chosen for different medical retrieval task.

### 2.1. Hand-Crafted Features

Hand-crafted features including texture features, keypoint-based features, local features, and global features are commonly used in CBMIR systems [5, 6, 8, 28, 29]. Jiang et al. [30] proposed a retrieval strategy that used mammographic region of interest (ROI) as query input, then retrieve breast tumor based on SIFT features. Caicedo et al. [31] used SIFT features to retrieve basal-cell carcinoma. Haas et al. [32] used SURF to capture the local texture of lung CTs for retrieval. Local Binary Patterns (LBPs) as local texture features were successfully used in ImageCLEFmed, 2D-Hela, and brain MRI retrieval tasks [33–35]. Xu et al. [36] proposed a corner-guided partial shape matching method that can dramatically increase the matching speed for spine X-ray image retrieval. Holistic features such as global GIST, global HOG, global color histogram, and moments were also used in medical image retrieval [37–41].

### 2.2. Learned Features Using Deep CNNs

In recent years, using features get through deep CNNs has achieved impressive results in generic image classification, object recognition, detection, retrieval, and other related tasks. But in the medical field, there is not much attention on exploring deep neural networks CBMIR task, partially because the amount of labeled medical images is typically limited. Qayyum et al. [10] proposed a CNN framework and trained the CNN on the medical image set they collected. Khatami et al. [9, 14] tried two retrieval strategies for medical image retrieval: the first method used one CNN model with transferred weights to shrink the search space and then used Radon projection to do similarity search. The second method employed multiple CNN models trained in a parallel way to get the shrunk search space. Bar et al. [42] used a pretrained CNN model from natural images for chest X-ray retrieval. Semedo and Magalhães [43] trained their CNN models on provided medical images in ImageCLEFmed 2016; they employed dropout and data augmentation to avoid overfitting. Hofmanninger and Langs [44] trained CNN using clinical routine images and radiology reports and carried out fine-tuning on current medical image retrieval task.

## 3. Methodology

**Figure 1:**
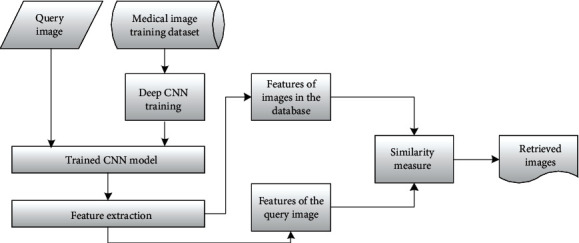
The proposed deep CNN-based content-based medical image retrieval flowchart.

There are pioneering studies that have been performed on deep CNNs for medical image retrieval and have shown promising results [9, 10, 14]; the problems of short of labeled images and highly imbalanced data distribution are still two main challenges for applying deep CNNs in medical image retrieval task [5]. There are also needs for more accurate and faster image retrieval methods for CBMIR [5]. To tackle these problems, in this work, we propose a multicomponent combined deep CNN framework for 2D medical image retrieval. The flowchart of content-based medical image retrieval is shown in Figure 1. This deep convolutional neural network is trained by a supervised learning way for classification and gets a concise feature vector for efficient nearest neighbor searching similar medical images. A brief description of the proposed framework is presented in the following sections.

### 3.1. Processing 2D Medical Image with Empirical Mode Decomposition (EMD)

**Figure 2:**
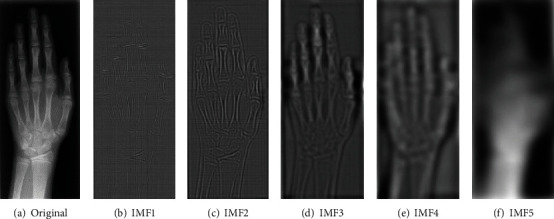
An example of a medical X-ray image is decomposed into five IMFs using empirical mode decomposition.

Empirical mode decomposition was originally introduced for the adaptive analysis of nonstationary and nonlinear time-domain signals and has become one of the most powerful tools for analyzing time-frequency (T-F) signal [45]. Then, EMD was extended to handle multidimensional data and acquired successful application in image tasks [46–49]. For image analysis, EMD is a fully data-adaptive multiresolution data analysis technique to decompose the multispatial resolution spatial-frequency-amplitude components of the image into a set of intrinsic mode functions (IMFs) [50, 51]. By advantage of the EMD principle, we can get multifrequency components (i.e., IMFs) of 2D medical images, and these frequencies are not predesigned; these frequencies can self-adapt to different content of an image. Thus, we acquire nonstationary and nonlinear multiresolution components of 2D medical images, which can provide supplementary information to commonly used spatial filter sets in image processing. EMD is implemented in an iterative process. First, a sifting process is used to find IMFs. Given a signal *x*(*t*), Equation (1) is the process to get one IMF.

(1) $$\begin{array}{c} x\left(t\right)-\overset{k}{\underset{i=1}{\sum }}m_{i}=h_{k}\Rightarrow h_{k}=c_{1}, \end{array}$$

where *m*_*i*_ is the local mean of the maxima and minima envelopes. These two envelopes are formed by connecting all local maxima or minima with a cubic spline. With the IMFs, the data *x*(*t*) can be decomposed by another sifting process:

(2) $$\begin{array}{c} x\left(t\right)-\overset{n}{\underset{j=1}{\sum }}c_{j}+r_{n}, \end{array}$$

where *c*_*j*_ (*j* = 1 to *n*) is the IMFs and *r*_*n*_ is the final residual component.  Figure 2 shows an example of a 2D X-ray image decomposed using EMD.

### 3.2. The Proposed Medical Image Retrieval Method

**Figure 3:**
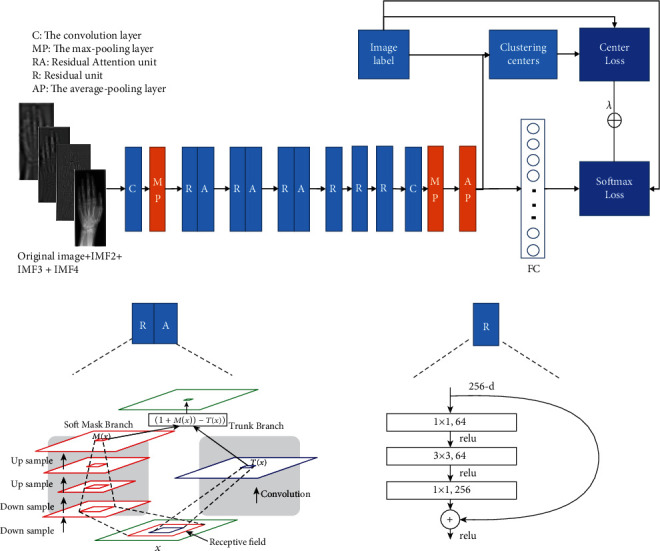
The proposed deep CNN framework for content-based medical image retrieval.

In this section, we introduce a deep CNN framework for medical image retrieval on a rather small dataset and with highly imbalanced data distribution. First, we discuss the network architecture employed in this work. Second, the supervision signal combining softmax loss function with center loss function to train deep CNN is discussed. Third, the training process is detailed. The proposed deep CNN framework is illustrated in Figure 3. For the input of the network, we employ original image and its IMF2, IMF3, and IMF4 components, because IMF1 contains mainly noise with quite high spatial frequency, and IMF5 contains the overall image intensity trend with very low spatial frequency. For medical image classification, IMF1 and IMF5 cannot provide useful structure information.

#### 3.2.1. The Network Architecture

The proposed deep CNN architecture employs Residual Attention Network (RAN) [27] as the backbone network. In RAN, mixed attention activation function is used for both spatial and channel attention. The attention mechanism was implemented as multiple attention modules, and each module consisted of a mask branch and a trunk branch, in which the mask branch was used to select good properties of original features and suppress noises from trunk features. Residual learning was introduced in the learning process of RAN; the mask branch was constructed as identical mapping. With the residual learning, Residual Attention Network can go very deep, and the training process was much efficient. For medical image retrieval task, the nearest-neighbor similarity search is the most common way used to rank retrieved images. If the length of vector used to compute the similarity between two compared medical images is too long, the retrieval process will be very time consuming and cannot be used in practice. Thus, a dimensionality reduction model is added to get concise while strong distinguishing features.  Table 1 details the CNN structure used in this work.

#### 3.2.2. Joint Loss Function

Wen et al. [52] firstly introduced center loss function in deep CNN for face recognition task. In their work, center loss function was linearly jointed with softmax loss function to form a mixture supervision signal to train deep CNN. These two loss functions that were used in conjunction with each other can achieve discriminative feature learning, that is, the deeply learned features contained intraclass compactness and interclass dispersion. Discriminative features are very suitable for medical image classification and retrieval task in which nearest-neighbor similarity search is most commonly used to accomplish the retrieve. Equation (3) formulates this joint loss function.

(3) $$\begin{array}{c} \text{Los}{\text{s}}_{\text{mixture}}=\text{Los}{\text{s}}_{\text{softmax}}+\lambda \text{Los}{\text{s}}_{\text{center}}-\overset{m}{\underset{i=1}{\sum }}\log\frac{e^{W_{y_{i}}^{T}x_{i}+b_{y_{i}}}}{\sum_{j=1}^{n}e^{W_{y_{i}}^{T}x_{i}+b_{y_{i}}}}+\frac{\lambda }{2}\overset{m}{\underset{i=1}{\sum }}{\left\|x_{i}-c_{y_{i}}\right\|}_{2}^{2}, \end{array}$$

where the left part is the original softmax loss and the right part is the center loss. The *c*_*yi*_ denotes the *y*_*i*_th class center in the form of a feature vector. The parameter *λ* is empirically set as 0.002 in this paper's experiments.

#### 3.2.3. Network Training Setting

As shown in Figure 3, the input of the network is the original medical image with its EMD components that contain IMF2, IMF3, and IMF4 got from EMD. The network training is developed and trained by using Keras on TensorFlow. The training processes are performed on a workstation with Ubuntu 18.04, having Intel(R) Xeon(R) Gold 6154 CPU with 256G RAM, and NVIDIA TITAN V graphic card with 12G RAM. Data argumentation and dropout are employed in the training process. The number of epochs is 500, the batch size is 16, the initial learning rate is 0.0001, and early stopping is on. When the network accuracy is not improved within 20 training iterations, the early stopping mechanism will be triggered. The 500 epoch setting is to make sure that in most cases, the network training is stopped by the early stopping mechanism.

## 4. Experimental Results

In this paper, the very challenging IRMA dataset is chosen to evaluate the proposed framework and compare with other methods reported in the literature. The proposed CNN model is evaluated in terms of classification performance and retrieval performance, respectively.

### 4.1. Database Description

**Figure 4:**
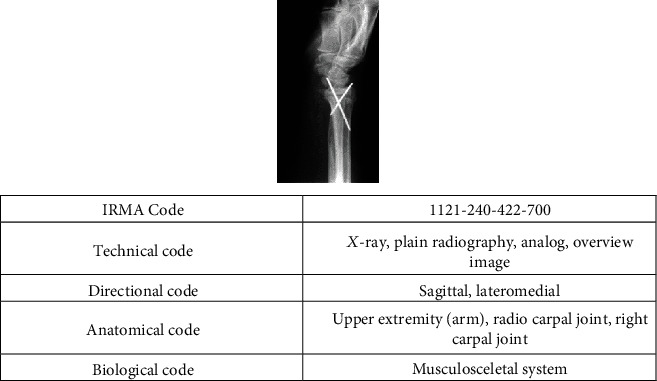
A sample image (arm) with the corresponding IRMA code.

IRMA (Image Retrieval in Medical Applications) database is a well-known medical image dataset for content-based medical image retrieval research, which was made by Aachen University of Technology (RWTH) [53]. This dataset was arbitrarily selected from a routine at the Department of Diagnostic Radiology, Aachen University of Technology. IRMA code is used to specify each image's class along four independent hierarchical axes: TTTT-DDD-AAA-BBB. In this code, T represents the technical code (imaging modality), D represents the directional code (body orientations), A represents the anatomical code (the body region examined), and B represents the biological code (the biological system examined). This dataset contains a total of 12,000 images divided into 116 classes, 11,000 image radiographs with known categories for training, and the rest 1000 radiographs as test.  Figure 4 illustrates a sample image with the corresponding IRMA code.

### 4.2. Classification Performance

#### 4.2.1. IRMA Error

ImageCLEF07 proposed the error evaluation procedure for IRMA Medical Image Annotation to calculate the retrieval error [54, 55]. The total IRMA error can be computed by the following formula:

(4) $$\begin{array}{c} \overset{I}{\underset{i=1}{\sum }}\frac{1}{b_{i}}\frac{1}{i}\delta \left(l_{i},{\hat{l}}_{i}\right)\text{ with }\delta \left(l_{i},{\hat{l}}_{i}\right)=\left\{\begin{array}{cc} 0, & \text{if }l_{j}={\hat{l}}_{j},\forall j\leq i,\\ 0.5, & \text{if }l_{j}=*,\exists j\leq i,\\ 1, & \text{if }l_{j}\neq {\hat{l}}_{j},\exists j\leq i. \end{array}\right. \end{array}$$

Here, *l*_1_^*I*^ = *l*_1_, *l*_2_, ⋯, *l*_*i*_, ⋯, *l*_*I*_ is the *correct* code (for one axis) of an image, and ${\hat{l}}_{1}^{I}={\hat{l}}_{1},{\hat{l}}_{2},\cdots ,{\hat{l}}_{i},\cdots ,{\hat{l}}_{I}$ is the *classified* code (for one axis) of an image. *I* is the depth of the tree to which the classification is specified. If there is an incorrect classification at position ${\hat{l}}_{I}$, all succeeding decisions will be considered as wrong decisions.

#### 4.2.2. Commonly Used Classification Performance Measure

To evaluate the performance of different methods for classification task, commonly used performance evaluation indicators include average precision (AP), average recall (AR), and F1 measure. These indicators are calculated as the following:

(5) $$\begin{array}{c} \text{AP}=\frac{1}{M}\overset{M}{\underset{i=1}{\sum }}\frac{{\text{TP}}_{i}}{{\text{TP}}_{i}+{\text{FP}}_{i}},\\ \text{AR}=\frac{1}{M}\overset{M}{\underset{i=1}{\sum }}\frac{{\text{TP}}_{i}}{{\text{TP}}_{i}+{\text{TN}}_{i}},\\ \text{F}1\text{ measure}=2\times \frac{\text{AP}\times \text{AR}}{\text{AP}+\text{AR}}, \end{array}$$

where TP is true positive, indicating the number of images correctly classified as class *k*; FP is false positive, indicating the number of images misclassified as class *k*; TN is true negative, indicating the number of images correctly classified as not class *k*; FN is false negative, indicating the number of images misclassified as not class *k*; and *M* means the total number of classes that is 116 IRMA classes in this paper. As the F1 measure is more sensitive to data distribution, it is a suitable measure for classification problems on imbalanced datasets [10].

#### 4.2.3. Classification Performance and Comparison

The performance of the proposed single-model framework for medical image classification is evaluated by the IRMA error and commonly used measures for image classification methods, which are detailed in Sections 4.2.1 and 4.2.2.  Table 2 compares the IRMA error got by the proposed framework and several deep CNN-based methods reported in the literature [9, 14].  Table 2 shows that with the fast development of the deep CNN technique, much better classification accuracy (i.e., lower IRMA error score) can be gotten by employing a more powerful CNN model as a backbone network. In terms of the IRMA error, our proposed framework gets a much lower score than referenced deep CNN-based methods reported in the literature.

Considering the relative lag of the technology applied on IRMA dataset and the rapid development of the deep CNNs in computer vision area, Table 3 compares the classification accuracy measures on the IRMA dataset including IRMA error, AP, AR, and F1 measure of the proposed method with various state-of-the-art deep CNNs including VGG [18], ResNet [19], and AttentionResNet [27] that have achieved a very high recognition score on large image dataset challenges (such as ImageNet [58] and CoCo [59]). Table 3 shows that the proposed framework performs better in classifying IRMA images. The proposed framework and the compared deep CNNs are trained under the same condition, that is, using the same training dataset, same image argumentation strategy, same number of epochs, same learning rate, and so on. For classification-based medical image retrieval, the retrieval performance depends entirely on the accuracy of classification, the higher classification accuracy means the better retrieval performance. As in Table 3, our proposed framework achieved the lowest IRMA error and the best F1 measure.

**Figure 5:**
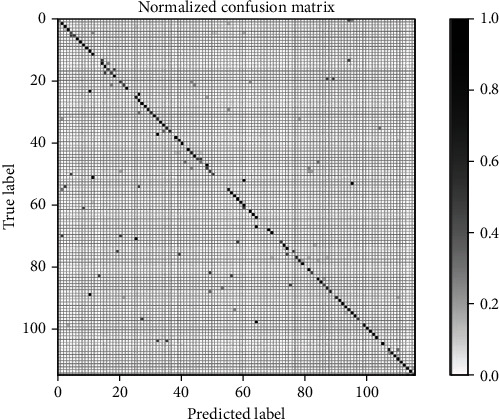
Confusion matrix of IRMA image classification with 116 classes using the proposed deep CNN.

The confusion matrix is shown in Figure 5, where most classes can be classified rightly. There are 38.2% classes with accuracy better than 90%, 51.2% classes with accuracy better than 80%, and 59.1% classes with accuracy better than 70%.

### 4.3. Retrieval Performance

#### 4.3.1. Retrieval Performance Measure

Precision and recall are two measures commonly used as retrieval performance evaluation measures [5].

(6) $$\begin{array}{c} P=\frac{\text{Numberofrelevantimagesretrieved}}{\text{Totalnumberofimagesretrieved}},\\ R=\frac{\text{Numberofrelevantimagesretrieved}}{\text{Totalnumberofrelevantimages}}. \end{array}$$

Besides precision and recall, mean average precision (MAP) is a very popular evaluation metric for algorithms that do search in medical image sets [5]. MAP combines precision and recall in one number. It is defined as the mean of average precision (AP) metric over all queries that can alleviate the bias during precision evaluation. The AP and mAP can be formulated as the following:

(7) $$\begin{array}{c} \text{AP}\left(q\right)=\frac{1}{N_{R}}\overset{N_{R}}{\underset{n=1}{\sum }}P_{q}\left(R_{n}\right), \end{array}$$

where *P*_*q*_(*R*_*n*_) is the precision value when the recall value is *R*_*n*_ and *N*_*R*_ indicates the top *N*_*R*_-ranked relevant images for the query image *q*.

(8) $$\begin{array}{c} \text{mAP}=\frac{1}{\left|Q\right|}\underset{q\in Q}{\sum }\text{A}\text{P}\left(q\right), \end{array}$$

where *Q* is the query image set and |*Q*| is the number of the query image set.

#### 4.3.2. Retrieval Performance and Comparison

In the proposed deep CNN framework, the feature vector for nearest-neighbor similarity searching of medical images is gotten from the last fully connected layer. For comparison, the proposed framework retrieval performance on the IRMA dataset is evaluated using both the IRMA error and the mean average precision (mAP). The calculation of the IRMA error in image retrieval follows the nearest-neighbor rule, that is, the query image's class label is determined by the most similar image returned in the retrieval process.  Table 4 compares the retrieval performance achieved by the proposed framework with the other methods reported in the literature [9, 14, 56, 57, 60, 61] on the IRMA dataset with the IRMA error. The proposed deep CNN framework gets the lowest IRMA error in nearest-neighbor similarity retrieval.  Table 5 compares the proposed framework with state-of-the-art deep CNNs on the IRMA error and mAP. For mAP, we test three usually used distance/similarity measures in image retrieval: Euclidean distance, Manhattan distance, and Cosine similarity, and the IRMA error is evaluated by using the best distance/similarity measure: Cosine similarity.  Table 5 shows that the proposed deep CNN framework gets the best mAP and the lowest IRMA error on these three distance/similarity measures and gets the highest score on Cosine similarity. In Table 5, we also list the vector length used for similarity retrieval. The feature vector for retrieval gotten from the proposed framework is just 32 dimensions that are much shorter than output vectors reported in literatures and state-of-the-art deep CNNs, which illustrate the great potential of our method to implement large-scare medical retrieval. Suppose **a** and **b** are two feature vectors representing two medical images, the three distance/similarity measures are formulated as the following:

(9) $$\begin{array}{c} \text{Euclidean distance}\left(a,b\right)=\sqrt{\overset{n}{\underset{i=1}{\sum }}{\left(a_{i}-b_{i}\right)}^{2}},\\ \text{Manhattan distance}\left(a,b\right)=\overset{n}{\underset{i=1}{\sum }}\left|a_{i}-b_{i}\right|,\\ \text{Cosine similarity}\left(a,b\right)=\frac{a∙b}{\left\|a\right\|∙\left\|b\right\|} \end{array}$$

**Figure 6:**
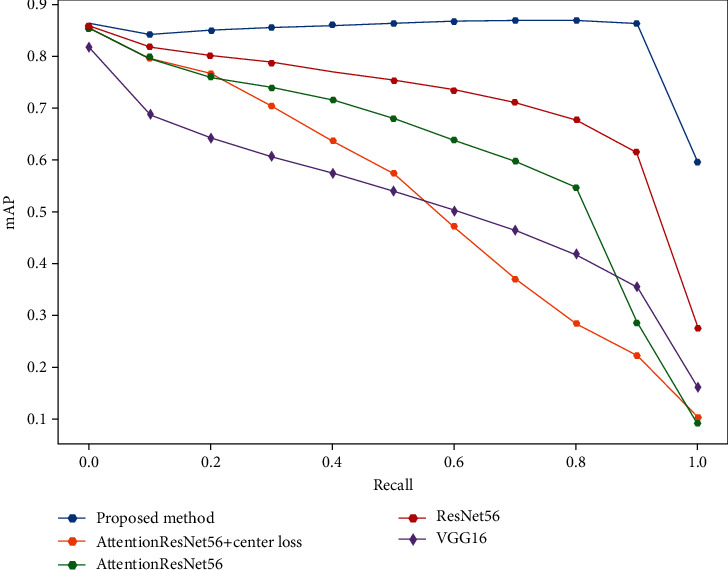
mAP vs. recall for medical retrieval on IRMA images.

Figure 6 summarizes the retrieval performance of the proposed framework and state-of-the-art deep CNNs by the mAP-recall curve. And all these curves are calculated using the Cosine similarity measure.

### 4.4. Performance Comparison with and without EMD Components

To illustrate the effect of EMD components, Table 6 details the classification and retrieval measures between the proposed framework and the state-of-the-art deep CNNs with and without using EMD components. The results show that with EMD components, we can get higher performance in both classification and retrieval applications. With EMD components, deep CNNs can consistently achieve better classification and retrieval performance than without EMD components except for VGG16 on the IRMA error. This may be because the ResNet backbone is deeper than VGG16, so the CNNs based on the ResNet backbone can effectively handle more image information.

## 5. Conclusions

This paper has proposed a deep convolutional neural network for medical image retrieval task. By training deep CNN with input medical image and its multifrequency components (i.e., IMFs get from empirical mode decomposition (EMD)) in a supervised classification way, we have got a scheme that is very suitable for similarity-based medical image retrieval. Using an imbalanced IRMA medical image dataset, the proposed framework has surpassed existing algorithms with the highest classification accuracy and lowest retrieval error. The concise and distinguishable feature vector output from the proposed deep CNN has also shown great potential to handle large-scale medical image retrieval. We intend to further examine CBMIR on other medical datasets, different modalities, and 3D volumetric applications.

## Figures and Tables

**Table 1:** The details of deep CNN used for medical image retrieval.

Layer name	Output size	Layer
Conv1	128 × 128	7 × 7, 64, stride 2
Max pooling	64 × 64	3 × 3 stride 2
Residual attention unit	64 × 64	$\left(\begin{array}{c} 1\times 1,64\\ 3\times 3,64\\ 1\times 1,256 \end{array}\right)\times 1$
		Attention mask block × 1
Residual attention unit	32 × 32	$\left(\begin{array}{c} 1\times 1,128\\ 3\times 3,128\\ 1\times 1,512 \end{array}\right)\times 1$
		Attention mask block × 1
Residual attention unit	16 × 16	$\left(\begin{array}{c} 1\times 1,256\\ 3\times 3,256\\ 1\times 1,1024 \end{array}\right)\times 1$
		Attention mask block × 1
Residual unit	8 × 8	$\left(\begin{array}{c} 1\times 1,512\\ 3\times 3,512\\ 1\times 1,2048 \end{array}\right)\times 3$
Conv2	8 × 8	3 × 3, 32, stride 1, padding: same
Max pooling	4 × 4	2 × 2 stride 1
Average pooling	1 × 1	4 × 4 stride 1
FC, softmax, Center loss	116	
Trunk depth	57	

**Table 2:** Comparison of our classification performance to other CNN reported in the literature for IRMA images. The IRMA error with ∗ was the best test value selected from the literature.

Methods	IRMA error
Proposed method	68.48
Parallel shrink CNN+Radon [9]	165.55^∗^
Sequential shrink CNN+LBP [14]	168.05^∗^
CNN+Radon [56]	210.35
CNNC+RBC [57]	224.13

**Table 3:** Comparison of classification performance of the proposed framework with other deep models and state-of-the-art on IRMA images.

Methods	IRMA error	AP	AR	F1 measure
VGG16 [18]	115.08	0.56	0.56	0.53
ResNet50 [19]	80.80	0.65	0.64	0.63
AttentionResNet56 [27]	76.83	0.65	0.66	0.64
AttentionResNet56+center loss [52]	73.85	0.68	0.66	0.66
Proposed method	68.48	0.67	0.67	0.66

**Table 4:** Comparison of our retrieval performance to other CNN reported in the literature for IRMA images. The proposed method used Cosine similarity measure to get the IRMA error in this table.

Methods	IRMA error
*Proposed method*	43.21
SVM+multiscale LBP [61]	146.55
Parallel shrink CNN+LBP, HOG, Radon [9]	165.55
Sequential shrink CNN+LBP [14]	168.05
TAUbiomed [60]	169.5
Diap [60]	178.93
CNN+Radon [56]	210.35
CNNC+RBC [57]	224.13
FEITIJS [60]	242.46
SuperPixel [57]	249.34
VPA [60]	261.16
SP-R [57]	311.8
MedGIFT [60]	317.53
SP-RBC [57]	356.57
IRMA [60]	359.29
MedGIFT [60]	420.91

**Table 5:** Comparison of retrieval performance of the proposed framework with other deep models and state-of-the-art on IRMA images. The IRMA error with ∗ was the best test value selected from the literature.

Methods	Vector dimension for similarity retrieval	IRMA error on Cosine similarity	mAP		
			Euclidean distance	Manhattan distance	Cosine similarity
Sequential shrink CNN+LBP [14]	8496	168.05^∗^	—	—	—
Parallel shrink CNN+LBP, HOG, Radon [9]	8496 (LBP)	165.55^∗^	—	—	—
	3528 (HOG)				
	1800 (Radon)				
SVM+multiscale LBP [61]	*N* subblocks × 4 × 4 × 1062 on SVM	146.55^∗^	—	—	—
VGG16 [18]	512	65.53	0.53	0.53	0.53
AttentionResNet56 [27]	2048	49.66	0.45	0.45	0.62
AttentionResNet56+center loss [52]	2048	47.76	0.35	0.35	0.53
ResNet50 [19]	2048	47.53	0.72	0.72	0.73
Proposed method	32	43.21	0.84	0.85	0.86

**Table 6:** Comparison of classification and retrieval performance of the proposed framework with other deep models and state-of-the-art on IRMA images with and without EMD components.

Methods	Classification				Retrieval (on Cosine similarity)			
	IRMA error		F1 measure		IRMA error		mAP	
	With EMD	Without EMD	With EMD	Without EMD	With EMD	Without EMD	With EMD	Without EMD
VGG16 [18]	115.08	98.29	0.53	0.52	65.53	57.18	0.53	0.54
ResNet50 [19]	80.8	92.62	0.63	0.55	47.53	62.2	0.73	0.70
AttentionResNet56 [27]	76.83	81.54	0.64	0.59	49.66	55.03	0.62	0.61
AttentionResNet56+center loss [52]	73.85	74.81	0.66	0.62	47.76	47.13	0.54	0.54
Proposed method	68.48	77.45	0.66	0.59	43.21	46.81	0.86	0.85

## Data Availability

The IRMA2007 data used to support the findings of this study are available in a public repository at http://publications.rwth-aachen.de/search?ln=en&cc=Dataset&sc=1&p=IRMA&f=&action_search=Search.
